# Infectious Keratitis: An Update on Role of Epigenetics

**DOI:** 10.3389/fimmu.2021.765890

**Published:** 2021-11-30

**Authors:** Sudhir Verma, Aastha Singh, Akhil Varshney, R. Arun Chandru, Manisha Acharya, Jyoti Rajput, Virender Singh Sangwan, Amit K. Tiwari, Tuhin Bhowmick, Anil Tiwari

**Affiliations:** ^1^ Department of Zoology, Deen Dayal Upadhyaya College (University of Delhi), New Delhi, India; ^2^ Department of Cornea and Uveitis, Dr. Shroff’s Charity Eye Hospital, New Delhi, India; ^3^ Pandorum Technologies Ltd., Bangalore Bioinnovation Centre, Bangalore, India; ^4^ Department of Pharmacology and Experimental Therapeutics, The University of Toledo, Toledo, OH, United States

**Keywords:** keratitis, epigenetics, methylation, histone modifications, infectious

## Abstract

Epigenetic mechanisms modulate gene expression and function without altering the base sequence of DNA. These reversible, heritable, and environment-influenced mechanisms generate various cell types during development and orchestrate the cellular responses to external stimuli by regulating the expression of genome. Also, the epigenetic modifications influence common pathological and physiological responses including inflammation, ischemia, neoplasia, aging and neurodegeneration etc. In recent past, the field of epigenetics has gained momentum and become an increasingly important area of biomedical research As far as eye is concerned, epigenetic mechanisms may play an important role in many complex diseases such as corneal dystrophy, cataract, glaucoma, diabetic retinopathy, ocular neoplasia, uveitis, and age-related macular degeneration. Focusing on the epigenetic mechanisms in ocular diseases may provide new understanding and insights into the pathogenesis of complex eye diseases and thus can aid in the development of novel treatments for these diseases. In the present review, we summarize the clinical perspective of infectious keratitis, role of epigenetics in infectious keratitis, therapeutic potential of epigenetic modifiers and the future perspective.

## Introduction

While the base sequence of the gene remains same, epigenetic mechanisms alter its expression and thus its function. This can happen *via* altered methylation of DNA, post-translational modifications of histones, introduction of non-coding RNAs, remodeling of the chromatin etc. Epigenetic mechanisms are known to play an important role in several pathophysiological conditions, including those of the ocular surface. Exposure of cornea to pathogens, leading to inflammation and keratitis, has previously shown to involve epigenetic mechanisms (1, 2).

Though our understanding of epigenetic mechanisms in keratitis has advanced to some extent in recent past, the clinical implications in terms of therapeutics and treatments are yet to be explored. Some of the examples of how the mechanistic understanding of epigenetics can potentially aid drug discovery in eye diseases can be: 1) Latent infection of HSV1 (Herpes Simplex Virus 1) in corneal cells can lead to persistent recurrence of keratitis (3). Knowing how to epigenetically reactivate the virus from its protective latent state could help in combating it *via* anti-HSV treatment. Knowing how to keep the virus in its latent state irrespective of epigenetic triggers could help in keeping the virus in a senile latent state without acute infection. 2) Fungal pathogens are known to vary their histone modifications to garner virulence and drug resistance. Down regulation of histone acetylation leads to increased inflammatory response in fungal keratitis, and histone deacetylase inhibitors could emerge as promising treatment (4). 3) In case of degenerative Keratoconus, the non-coding RNAs have potential to affect the expression of about 1000 genes (5).

Hence, understanding the epigenetic networks and interactions can possibly help in the early detection of diseases of the ocular surface and also lead to the development of novel therapeutic approaches (6). In the present review, we briefly summarize the role of epigenetics in ocular diseases followed by specifically focusing the infectious keratitis and epigenetic changes from a diagnostic and therapeutic perspective which can be possibly translated into novel therapies in the near future.

## Epigenetics and It’s Role in Ocular Diseases

‘Epigenetics’ refers to the heritable, reversible and environment-influenced mechanisms that affect the gene expression without altering the underlying DNA sequences (1, 7, 8). The term was initially used to refer to the complex interactions between the genome and the environment, involved in the development and differentiation of distinct cell lineages in higher organisms (9, 10). The epigenetic mechanisms that potentially mediate this dynamic interaction between the genes and the environment comprise of DNA methylation, chromatin remodeling, histone variants, post-translational modifications of histone and deployment of non-coding RNAs (11). Various factors contribute in the acquisition, maintenance and inheritance of diverse epigenetic modifications.

The modification of DNA and histone tails regulates the structure of chromatin and accessibility of DNA to transcriptional machinery. The principal epigenetic modification found in DNA is covalent attachment of methyl group by DNA methyl-transferase (DNMTs) enzymes at C5 position of cytosine residues in CpG dinucleotide sequence, which is a mark for transcriptional repression (12). On the other hand, histones can restructure the chromatin in transcriptionally permissive or restrictive states by undergoing diverse post-translational modifications such as acetylation, methylation, phosphorylation, ubiquitination etc. (13). These modifications are written, read and erased by a variety of histone modifying enzymes. The type, site, combination and the extent of histone modification adds the complexity of histone code (13–15). Besides modifications of DNA and histones, long non-coding RNAs (IncRNAs), micro RNAs (miRNAs), small inhibitory RNAs (siRNAs) and piwi interacting RNA (piRNAs) can also mediate transcriptional silencing as reviewed by Wei et al. (2017) (16). Also, the temporal and spatial regulation of transcription is regulated by ATP-dependent chromatin remodelers that re-configure the nucleosomes in response to environmental and developmental cues (17). These chromatin remodeling enzymes have been classified in subfamilies i.e. Switch/sucrose non-fermentable (SWI/SNF), imitation switch (ISWI), chromodomain helicase DNA-binding (CHD) and INO80 (18). Additionally, the replacement of canonical histones with the variants also leads to diversity of nucleosomes’ structure and function. The histone variants, their chaperones/remodeler machineries and linkage to various diseases have been extensively reviewed recently (19, 20).

**Figure 1:**
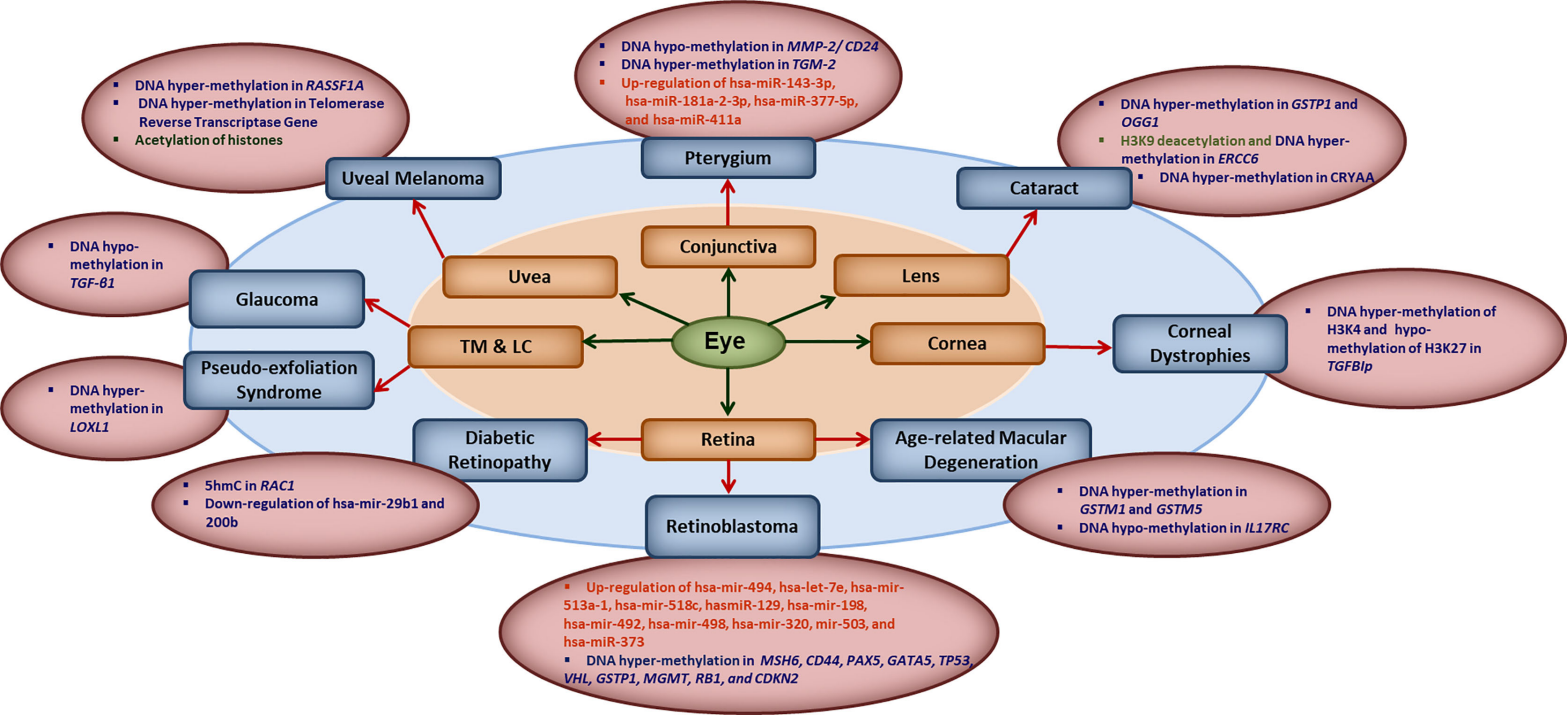
Key epigenetic modifications in common ocular diseases: Diverse epigenetic modifications are associated with the common ocular diseases occurring in different parts of the eye. MMP2, matrix metalloproteinase 2; CD24, CD24 molecule; TGM2, transglutaminase 2; hsa-miR, human microRNA; GSTP1, pi-class glutathione-S-transferase; OGG1, 8-oxoguanine DNA glycosylase 1; ERCC6, excision repair 6 chromatin remodeling factor; CRYAA, crystalline Alpha-A; TGFBIp, transforming growth factor β- induced; GSTM1/5, glutathione S-transferase isoform mu1/mu5; IL17RC, interleukin-17 receptor C; MSH6, mutS homolog 6; CD44, cluster of differentiation 44; PAX5, paired box 5; GATA5, GATA binding protein 5; TP53, tumor protein 53; VHL, Von Hippel-Lindau gene; GSTP1, glutathione S-transferase pi-1; MGMT, methylguanine methyltransferase; RB1, retinoblastoma 1; CDKN2, cyclin-dependent kinase inhibitor 2; 5hmC, 5-hydroxymethyl cytosine; RAC1, rac family small GTPase 1; LOXL1, lysyl oxidase-like 1; TGF-β1, transforming growth factor-β1; RASSF1A, RAS association domain family 1A gene; TM, trabecular meshwork; LC, the lamina cribrosa.

The human epigenome gets influenced by various factors such as diet, age, environmental factors, smoking and the infections. Evidences are growing that natural infections alter the epigenome by modulating the immune response and longitudinal disease risk. Most of the studies in infection-induced epigenetic changes have been done with respect to carcinogenic microbes and very less is known about epigenetic effects of non-carcinogenic microbial infections (21). Even less is known about the role of epigenetics in ocular infections and diseases. In this section, we briefly summarize the reported literature on involvement of different epigenetic mechanisms in ocular diseases (**Figure 1**). Some of the common eye disorders where role of epigenetic mechanism have been revealed, include retinoblastoma, diabetic retinopathy, age-related macular degeneration (AMD), glaucoma, cataract, keratoconus, corneal dystrophies, pterygium, keratitis etc. (2, 6, 22, 23), which affect different parts of the eye as shown in **Figure 1**. From the point of view of type of epigenetic mechanism involved, a large number of genes have been reported to undergo hyper- or hypo-methylation in different eye diseases such as: *MMP-2/CD24* and *TGM-2* in Pterygium; *GSTP1, OGG1*, *ERCC6* and CRYAA in cataract; *TGFBIp* in corneal dystrophies; *GSTM1, GSTM5* and *IL17RC* in *AMD, MSH6, CD44, PAX5, GATA5, TP53,VHL, GSTP1, MGMT, RB1* and *CDKN2* in retinoblastoma; *RAC1* in diabetic retinopathy; *LOXL1* in pseudo-exfoliation syndrome; *TGF-β1* in glaucoma; *RASSF1A* and telomerase reverse transcriptase gene in uveal melanoma etc. The level of various micro-RNAs has also been reported to be altered in different eye diseases such as up-regulation of hsa-miR-143-3p, hsa-miR-181a-2-3p, hsa-miR-377-5p and hsa-miR-411a in Pterygium; up-regulation of hsa-mir-494, hsa-let-7e, hsa-mir-513a-1, hsa-mir-518c, hasmiR-129, hsa-mir-198, hsa-mir-492, hsa-mir-498, hsa-mir-320, mir-503, and hsa-miR-373 in retinoblastoma; and down-regulation of hsa-mir-29b1 and 200b in diabetic retinopathy etc. Besides these, histone modifications also seem to play important role as revealed by H3K9 deacetylation in *ERCC6* in cataract; global histone acetylation in uveal melanoma etc.

These studies definitely attract our attention towards possible involvement of different epigenetic mechanism in induction, execution and promotion of various eye disorders with an opportunity to explore this area for better diagnostic and therapeutic targets. Having convinced with that, we next focus on infectious keratitis as another important eye disease of global concern and the epigenetic mechanisms involved in it.

## Infectious Keratitis – Types, Clinical Features, and Management

**Figure 2:**
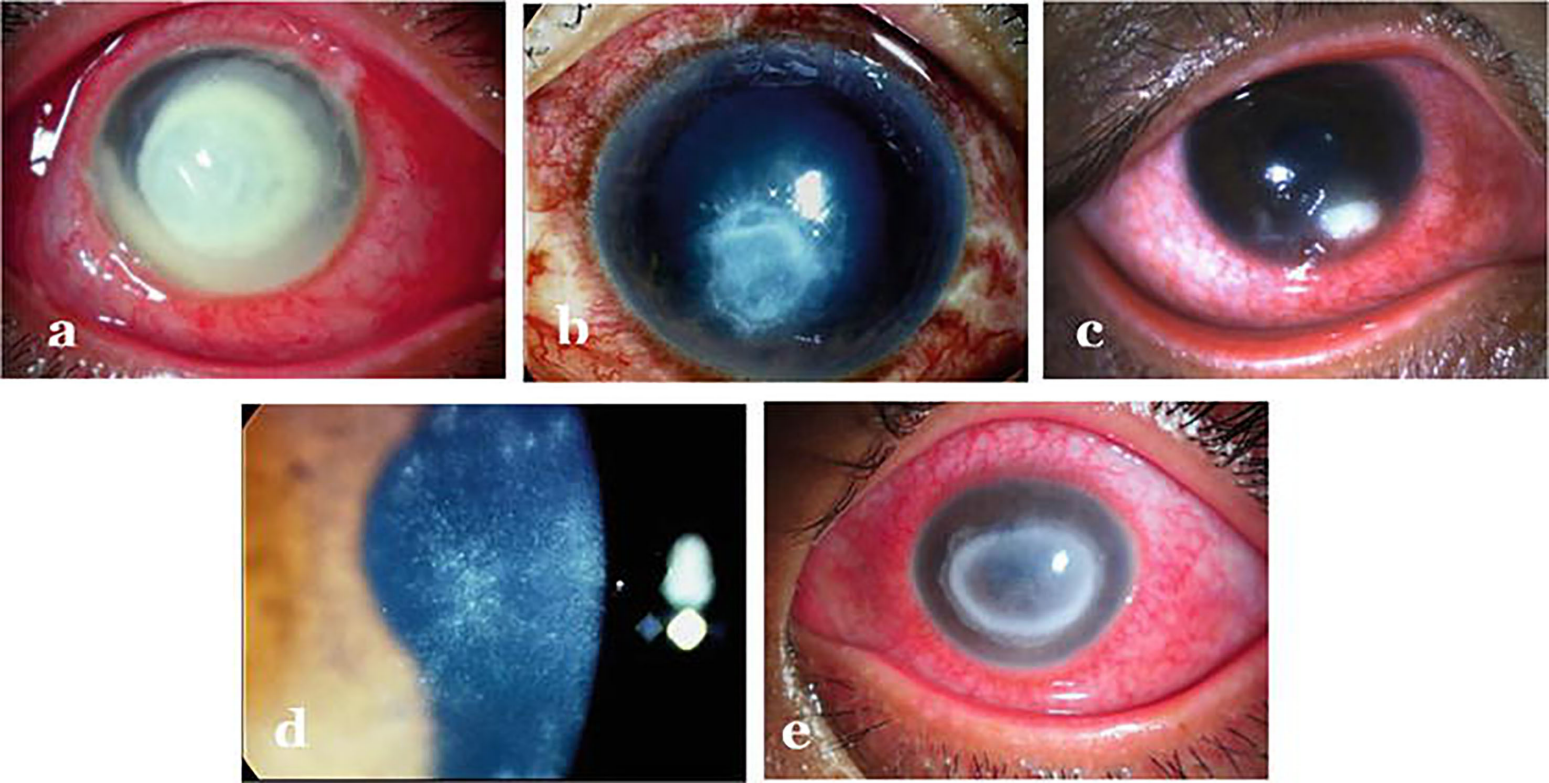
Infectious keratitis caused by different agents: **(A)** Bacterial keratitis **(B)** Fungal keratitis **(C)** Herpes necrotizing stromal keratitis **(D)** Early *Acanthamoeba* keratitis **(E)** Late *Acanthamoeba* keratitis. Adopted from https://www.intechopen.com/chapters/69696 under Creative Commons Attribution 3.0 License.

Keratitis refers to inflammation of cornea i.e. clear tissue in the front of eye covering pupil and iris. Depending on the causative agent, keratitis is broadly classified as non-infectious or infectious. The non-infectious keratitis results due to injury, exposure to intense sunlight, dry eyes, weak immunity etc. The infectious one on the other hand is caused by variety of microbes i.e. bacteria, viruses, fungi, parasites etc. (**Figure 2**). The cornea, which remains protected anatomically by the eyelids, a healthy tear film & its protective factors, an active lacrimal drainage system and a tenacious epithelial cover gets inflamed if any of these protective factors is breached by microbial invasion. Infectious keratitis or corneal ulceration is traditionally described as a defect in the corneal epithelium, accompanied with infiltration and inflammation. Active keratitis and its sequelae in the form of corneal perforation or scarring can cause significant morbidity and even complete vision loss (24).

- Infectious keratitis is the most common cause of corneal blindness in both developing and developed world (25). Estimated incidence of infectious keratitis is reported to be ranging from 2.5 to 799 per 100,000 population-year, depending on the study design and geographical location (26). A higher rate of infectious keratitis in under-resourced countries and a wide variation in prevalence of causative organisms and thus, the frequency of microbial keratitis has been reported from different parts of the world (26, 27)). These variations have been widely attributed to poor environmental and personal hygiene, lack of awareness and healthcare, agriculture and work-related trauma etc. But variations are also expected to exist in terms of diets and metabolites in different geographical & socio-economical regions. Thus, the gut microbiome-host immune interactions along with ocular surfaces microbiota also vary which in turn indicate the involvement of epigenetics in varied induction and promotion of infectious keratitis. Dysbiosis i.e. imbalance in gut microbiome has already been reported to be associated with bacterial keratitis (28).
- Bacterial keratitis is the commonest form of infectious keratitis globally with incidence ranging from 50 to 60% (29). The potential risk factors for bacterial keratitis include contact lenses, aqueous tear deficiencies, trauma, decreased immunologic defenses, eyelid alterations or malposition, neurotrophic keratopathy, topical corticoid medications and surgery (30). The common corneal ulcers causing bacteria are *Staphylococcus* spp., *Streptococcus* spp., Enterobacteriaceae (including *Serratia*, Klebsiella*, *Enterobacter and *Proteus*) and *Pseudomonas* spp. Fungal keratitis on the other hand is seen in 6-30% of cases and mainly caused by *Aspergillus* and *Fusarium species* (31). Incidence of HSV (Viral keratitis) and *Acanthamoeba* keratitis are 15-40% and 0-5% respectively (32). Also, mixed infections comprising of infliction by more than one organism is also seen in 2-15% of patients (29).

Clinically, the patient presents with complaints of redness, pain, watering, diminished vision and intolerance to light referred to as photophobia. As far as diagnosis is concerned, distinguishing features such as - feathery borders and fixed hypopyon in fungal keratitis, diminished corneal sensations in viral keratitis and ring infiltration in *acanthamoeba* keratitis are used for clinical diagnosis (29) (**Figure 2**). Confirmatory diagnosis of infective keratitis is made conventionally on microbiological examination which includes smear examination, culture and polymerase chain reaction (PCR) evaluation (33). *Acanthamoeba* cysts have additionally been reported to be detected on confocal microscopy (34). However, culture negative keratitis poses significant problem to clinicians. Next generation sequencing (NGS) can help in diagnostic accuracy of infectious keratitis especially in culture-negative cases. Tian et al. (35) have analyzed differentially expressed genes (DEGs) in bacterial and fungal keratitis. A total of 148 DEGs were found only in bacterial keratitis and 50 DEGs only in fungal keratitis. Besides, they also identified 117 co-expressed gene pairs among bacterial keratitis DEGs and 87 pairs among fungal keratitis DEGs. Also, a total of nine biological pathways and seven KEGG pathways were screened and found that TLR4* *is the representative DEG specific to bacterial keratitis, and SOD2 is the representative DEG specific to fungal keratitis, and hence can be used as promising candidate genes to distinguish between bacterial and fungal keratitis. Thus, at molecular level, genes can be quantified for identifying the causative agent for specific therapeutic outcomes. Though NGS can undoubtedly provide better insights about the ocular surface microbiome in pathophysiological circumstances, but it is not clear whether these can be effectively used to determine etiology of infection or antibiotic sensitivity. As far as management of keratitis is concerned, antimicrobial agents (36–39) besides collagen crosslinking (40–42) have been the mainstay for therapy. But, in the light of differential gene expression, specific pathways involved and eye-microbiota-immune interaction; it will be interesting to explore the epigenetic mechanisms involved so that specific epidrugs can be identified for treating infectious keratitis caused by particular type of microbe.

## Epigenetics of Infectious Keratitis

Bacterial keratitis or often referred as ‘corneal ulcer’, is the most common form of infectious keratitis. Bacteria can induce inflammatory cascade through the interaction of pathogen associated molecular patterns (PAMP) with Toll-like receptors (TLR) expressed on corneal and conjunctival epithelial cells and subsequently activate the mitogen-activated protein kinases (MAPK) cascade and NF-κB, leading to increased production of inflammatory cytokines. Importantly, the production of inflammatory cytokines is under the control of epigenetic factors like histone acetylation/deacetylation (43). However, very little is known about epigenetic mechanisms in bacterial keratitis. Nonetheless we might be able to learn from pathogenesis of bacterial infections in other systems where the role of epigenetic factors has been investigated and can be extrapolated in the field of bacterial keratitis. In cardiomyocytes, lipopolysaccharide (LPS), a component of the bacterial cell wall, was found to increase histone deacetylase (HDAC) activity. Since HDAC3 regulates TNF production, its inhibition decreases LPS-stimulated tumor necrosis factor (TNF) expression caused by the accumulation of nuclear factor kappa-B (NF-κB)/p65 at the TNF promoter (44, 45).

Herpetic keratitis is another common infectious corneal disease, caused by Herpes simplex virus 1 (HSV1). HSV1 infects corneal epithelial cells and sensory neurons thereby establishing latent infection, leading to recurrence of HSV1 in the cornea upon activation of virus under the influence of various stimulatory factors. Only the latency associated transcript (LAT) remains persistently expressed and lytic genes remain transcriptionally repressed, thereby maintaining the latency phase. Therefore, in order to understand and treat HSV infection, it is critical to understand the mechanism by which HSV1 is maintained in latent phase and how HSV1 is activated. The division of active and inactive genome has been shown to have epigenetic control. Histone modification for active transcription i.e. di-methylation of H3K4 and acetylation of H3K9 and H3K14 in LAT region and for inactive transcription i.e. trimethylation of H3K27 along with macro H2A histone variant have been reported to execute this. Moreover, chromatin insulators seem to separate the epigenetic domains of LAT and lytic genes. Abrogation of these insulators and CTCF (the protein that binds vertebrate insulators) binding possibly pave the way for transition from lytic to lysogenic phase (3, 46). Additionally, in neuronal cells, HDAC inhibitors (trichostatin-A) have been reported to reactivate the HSV1 infection in LAT-independent manner too (47).

Neurotrophic keratitis, also known as neurotrophic keratopathy, is a degenerative corneal disease caused by damage of trigeminal innervation. This damage to corneal innervation (from the trigeminal nucleus to the corneal nerve endings at different levels on the fifth cranial nerve) can be caused by various ocular surface disease, systemic diseases and central or peripheral nervous damages (48, 49). Though neurotrophic keratitis does not come under infectious keratitis directly but is most commonly induced by HSV (herpetic keratitis), the neurotrophic virus (50). With reactivation of latency by various stimulatory factors, virus travels back to the corneal epithelium along the axon and causes damage to corneal nerve with a severe reduction of sub-basal nerve plexus density with resultant diminished corneal sensation or corneal anesthesia (51). Thus, the epigenetic mechanisms involved in herpetic keratitis can be extrapolated to understand and manage neurotrophic keratitis too.

The pathogenesis of fungal keratitis remains poorly understood and therefore, its treatment is also yet to be explored more, especially from epigenetic perspective. However, Xiaohua Li et al. (4), have recently reported the attenuation of fungal keratitis in mice by histone deacetylase inhibitor, suberoylanilide hydroxamic acid (SAHA). It implies histone acetylation-deacetylation as potentially important target for understanding the fungal keratitis and expedite research in this area for better diagnosis and therapeutics. Additionally, a comprehensive human corneal miRNA expression profile and associated regulatory role in fungal keratitis has been reported (52) which again indicate the possible role of epigenetics in fungal keratitis as well.

*Acanthamoeba* keratitis, caused by *Acanthamoeba castellanii* remains a challenge to treat because of encystation. Even a single cyst in the tissue can cause re-infection and therefore, an effective strategy must inhibit cyst formation as well besides killing the pathogen. Epigenetic modification of the genes and proteins involved in initiation & maintenance of cyst and transition from cyst to active form can thus be a potential target for the same. Expression of encystation-mediating cyst-specific cysteine proteinase (CCSP) gene is regulated by DNA methylation (53). Similarly, silent-information regulator 2 like protein (SIR-2), which is a nicotinamide adenine dinucleotide-dependent deacetylase plays role in growth and encystation of *Acanthamoeba* (54). Though there are no direct reports available for involvement of epigenetics in *Acanthamoeba* keratitis, these reports suggest that epigenetic mechanisms play vital roles in Acanthamoeba physiology and pathology and thus, can be explored for medical purposes.

## Epigenetics Modifiers as a Potential Therapeutic Molecule

Dysregulated epigenetics is involved in a wide range of diseases like cancer, blood disorders, neurological and neurodegenerative disorders, and respiratory disorders (55). The ability to reverse the epigenetic modifications makes them an attractive druggable target (56). The changes in epigenetic landscape can be used as diagnostic markers as well as therapeutic targets in both invasive and non-invasive samples (57). Besides pharmacokinetic effects of epigenetic-based drugs, one can also consider the pharmacodynamics effects of epigenetics. The pharmacoepigenetics, the study of the epigenetic basis for variations in drug response is a growing field which highlights that the genes encoding drug-metabolizing enzymes, nuclear receptors, drug transporters etc. are under epigenetic control and thus, can affect the pharmacodynamics of drugs (58).

The epigenetic modifiers fall into three main categories i.e. writers (ones that mark DNA and histones with chemical groups), readers (which read those marks) and erasers (which remove these marks). All three have been targeted for developing epigenetic-based drugs. Besides having precise knowledge about molecular targets and the mechanism of action involved, the demonstration of efficacy is what ultimately matters for drug. The epigenetic-based drugs are a reality now, but we need to be aware that it’s a recent development and there are concerns about specificity, adversity, best schedule, ideal dosing, downstream effectors etc. Nevertheless, there are many epi-drugs which are either already approved by the U.S. Food and Drug Administration (FDA) or they are at advanced stages of approval. But most of them are for cancers. Presently, epi-drugs in three epigenetic target classes (i.e. DNMT, HDAC and EZH2 inhibitors) have been approved for the treatment of diverse malignancies (59). So far, there is no approved epigenetic-based biomarker and drug by the U.S. FDA for ocular diseases, particularly keratitis.

However, some of the recent studies have demonstrated promising therapeutic potential of epigenetics in infectious keratitis, which develops our hope that we might have epi-drugs for ocular diseases soon as well. For example, Sivakarthik Varanasi et al. (60), have shown that 5-azacytidine (Aza; a cytosine analog), a DNA methyltransferase inhibitor, inhibits the progression of herpatic keratitis and limits the HSV-1-induced ocular inflammatory lesions by enhancing regulatory T-cell function. Similarly, attenuation of fungal keratitis in mice by histone deacetylase inhibitor, suberoylanilide hydroxamic acid (SAHA) has been recently reported by Xiaohua Li et al. (4),. Also, Hae-Ahm Lee et al. (61) have recently shown that histone deacetylase inhibitors MPK472 and KSK64 can be potential therapeutic targets for *Acanthamoeba* keratitis, which otherwise is difficult to treat because of cyst formation. These HDACs inhibit the encystation of A*canthamoeba* and have low cytopathic effects on human corneal epithelial cells, and therefore can be promising epidrugs for *Acanthamoeba* induced keratitis.

Using combinations of epigenetic modifiers can also be an important strategy in reducing inflammation and/or disease, for example, a single dose of combinatorial administration of as 5-Aza-2-deoxycytidine (Aza) and trichostatin A (TSA) (Aza+TSA) after the onset of acute lung injury (ALI) has been found to be an effective method to attenuate lung vascular hyper permeability and inflammatory lung injury (62).

In context of viral diseases, epigenetic modifiers in the latency period of infection can be controlled in two steps i) Shock and kill strategy-using epigenetic modifiers to revoke the expression of virus and use anti-viral drugs to decrease viral load and ii) block and lock strategy- using epigenetic modifiers permanently silencing the latent virus (63). Similar strategy could be potential used in case of ocular inflammatory disorders and infectious keratitis complications (64).

## Conclusions and Future Perspective

Disease’s state represents an accelerated situation of tissue damage and aging. The role of epigenetics in maintaining normal development and function is reflected by the facts that many diseases develop when aberrant type of epigenetic footprints are introduced or are added at the wrong time or at the wrong place. DNA methylation, histone modifications and nucleosome positioning are generally used as a biomarker of tissue aging, it is not just marking time like a clock on the wall but “actually controlling the time-speed within cells” (65).

Age-associated DNA damage drives erroneous distribution of proteins at various cellular compartments. In case of epigenomic machinery it may cause unwanted genes to switch on/off associated with various diseases/degenerated state. In ocular context, epigenetic reprogramming has shown promising results in promoting optic nerve regeneration, reversal of vision loss in glaucoma, and reversal of vision loss in aging animals.

Corneal keratitis specifically neurotrophic keratitis is a condition of nerve degeneration. Manipulating epigenetic clock, thereby promoting nerve innervations could be one of the strategies to induce diseases clearance and healing. One approach will be to rewire the epigenetic memory rather than totally erasing it, by either controlling the dose, time exposure or different permutation and combinations modulating of Yamanaka factors. The reversal state can be driven by changing landscape of the tissue associated with earlier time stamp, thereby triggering local tissue regeneration (66–69). With the evolution of therapeutics, we have moved from small molecule drugs like aspirin to large molecule biologist such as insulin now moving into multicomponent system therapeutics, which may enable epigenetic reprogramming to induce targeted regeneration in the tissue of interest. Degeneration changes in a tissue associated with disease or aging are often linked with system level changes in functional gene clusters such as inflammation, fibrosis, neurodegeneration and vascular defects. Regeneration can be often looked at reversal of cell state with projections along these functional axes and changing the epigenetic state and effective time stamp and potential therapeutics using multiple factors

It is very essential to understand the epigenetic machinery and diseases specific function of its component to design and develop targeted epigenetic therapy. Importantly, it is critical to know the specific inhibitors other than the widely used pan inhibitors in clinical trials and further explore their roles in regulating specific gene expression in a more defined fashion during infection development and progression.

In the recent years, epigenetic studies advancement has provided novel insights and has significantly increased our knowledge about the interactions between pathogens, cellular factors, histones, and nonhistones modifying enzymes. As most of the epigenetics modifications are reversible, rewiring this complex machinery could be critical in determining the infection and also subsequent recovery. In case of viral keratitis, it is important to permanently maintain the virus in latency by erasing its reactivation epigenetic memory, so that the reactivation could be bypassed. Alternatively, using the epigenetic modifiers that targets the host rather than the pathogens could be helpful in tackling the complications of drug induced resistance in bacteria and viruses. In addition, addressing the role of the less studied post-translational modifications such as phosphorylation or sumoylation can shed light on new aspects of the dynamic host-pathogen interplay in case of infectious keratitis. Altogether, new therapeutic approaches are actively needed to treat infectious keratitis especially for viral infections and understanding the epigenetics of infectious keratitis and thereby repurposing drugs targeting epigenetic players could lead to major therapeutic breakthroughs in the treatment of ocular keratitis.

There are few important considerations to be taken into account, it is important to decrease the risk of epigenetic instability and abnormalities that could result due to continuous use of wide spectrum inhibitors over long-term. Therefore, it is very important to focus our research on identifying diseases specific inhibitors rather than global nonspecific inhibitors.

## Author Contributions

Conception, design, review of literature, writing, compiling, editing, and reviewing of the manuscript have been done by AT and TB. Conception, design, editing, and reviewing of the manuscript and final approval have been done by SV, AS, and AV. review of literature, writing, compiling, and editing have been done by RC, JR, MA, AT, and VS. The manuscript has the final approval of all the authors. The first three authors (SV, AS, and AV) have contributed equally and share the first authorship.

## Funding

AT thanks Department of Biotechnology (DBT), Government of India for Ramalingaswami Fellowship. AV thanks Department of Science and Technology (DST), Government of India, for Ramanujan Fellowship.

## Conflict of Interest

Author TB, AC, and JR are employed by the company Pandorum Technologies Ltd.

The remaining authors declare that the research was conducted in the absence of any commercial or financial relationships that could be construed as a potential conflict of interest.

## Publisher’s Note

All claims expressed in this article are solely those of the authors and do not necessarily represent those of their affiliated organizations, or those of the publisher, the editors and the reviewers. Any product that may be evaluated in this article, or claim that may be made by its manufacturer, is not guaranteed or endorsed by the publisher.
